# Clinicopathologic risk factors for post-operative complications after enucleation of pancreatic neoplasms

**DOI:** 10.1186/s12957-025-03920-0

**Published:** 2025-07-22

**Authors:** Isheeta Madeka, Steven Yi, Julia Evans, David Baek, Haresh V. Naringrekar, Harish Lavu, Charles J. Yeo, Avinoam Nevler, Wilbur B. Bowne

**Affiliations:** 1 Jefferson Pancreatic, Biliary and Related Cancer Center, Sidney Kimmel Comprehensive Cancer Center, Thomas Jefferson University Hospital, 132 South 10, Philadelphia, PA 19107 USA; 2 Sidney Kimmel Medical College, Thomas Jefferson University, Philadelphia, PA 19107 USA; 3 Department of Radiology, Thomas Jefferson University Hospital, 132 South 10th Street, Philadelphia, PA 19107 USA

**Keywords:** Pancreas, Neoplasms, Enucleation, Parenchymal sparing, Postoperative, Fistula, Complications

## Abstract

**Background:**

Pancreatic enucleation is a parenchymal-sparing procedure used for highly select patients with pancreatic neoplasms. We aim to utilize a multi-institutional health research network platform (TriNetX) and a single, high-volume center to assess complications and identify risk factors associated with post-operative pancreatic fistulas (POPF) after pancreatic enucleation.

**Methods:**

A two-tiered retrospective study was conducted. We identified 423 patients from TriNetX, and 34 patients from a single-institution IRB-approved database who underwent pancreatic enucleation between 2004–2025 and 2012–2023, respectively. Univariate and multivariate analyses were performed to determine risk factors associated with post-operative complications and occurrence of POPFs.

**Results:**

In the TriNetX cohort, 128 (30.3%) experienced postoperative complications after pancreatic enucleation. On univariate analysis, hyperlipidemia (HLD) (OR = 2.37), gastroesophageal reflux disease (GERD) (OR = 3.87), acute pancreatitis (OR = 8.28), chronic pancreatitis (OR = 4.76), nicotine dependence (OR = 2.36), ascites (OR = 6.49), deep vein thrombosis (DVT), pulmonary embolism (PE), and thrombophlebitis (OR = 2.95), and body mass index (BMI) ≥ 25 (OR = 1.56) were identified as significant risk factors. On multivariate analysis, acute pancreatitis (HR = 1.64), chronic pancreatitis (HR = 1.78), ascites (HR = 2.96), DVT, PE and thrombophlebitis (HR = 1.74) remained significant.

In our single-institution enucleation cohort, 8 patients had a POPF (23.5%). The measured distance from the neoplasm to the main pancreatic duct (MPD) was significantly shorter in patients who developed POPF (2.8 vs 6.5 mm, *P* < 0.05). ROC analysis determined that shorter distance from the MPD was predictive of POPF occurrence (AUC = 0.79, *p* < 0.005). Increased estimated blood loss was also associated with POPF (*p* < 0.01).

**Conclusion:**

Our study identifies clinicopathologic risk factors associated with post-operative complications and POPF after pancreatic enucleation. The distance from the neoplasm to the MPD appears to be a key component of decision-making in the development of POPF.

**Supplementary Information:**

The online version contains supplementary material available at 10.1186/s12957-025-03920-0.

## Background

Benign and low-risk pancreatic tumors were traditionally resected with standard pancreatectomies. However, resection of pancreatic parenchyma increases the risk of endocrine and exocrine pancreatic insufficiency, with morbidity rates approaching 50%. Over recent decades, pancreatic enucleation has emerged as an alternative treatment strategy to preserve pancreatic parenchyma that consequently lowers the risk of endocrine and exocrine insufficiency [1]. Several studies describe shorter operative times, fewer post-operative complications, and faster post-operative recovery in patients who undergo enucleation compared to standard pancreatic resection [2–4].

Pancreatic enucleation is considered in select patients with low-risk neoplasms such as insulinoma, nonfunctioning pancreatic neuroendocrine tumors (NF-pNETs), serous (SCN) and mucinous cystadenoma (MCN), solid papillary cystic neoplasms (SPCN) and branch duct type intraductal papillary mucinous cystic neoplasms (BD-IPMN) [5]. Despite enucleation’s ability to preserve the pancreas’s physiological function and potential for improved short- and long-term clinical outcomes, this procedure has been associated with a high occurrence of post-operative pancreatic fistulas (POPFs). Recent studies cite an incidence of POPF between 10 to 45% after enucleation[6]. Risk factors predictive of post-operative complications, specifically fistula formation are largely derived from limited studies and small patient cohorts [2–4]. Evidence-based data to identify clinicopathologic factors to better determine optimal candidates for pancreatic enucleation is warranted.

We therefore aim to perform a two-tiered retrospective study to identify clinicopathologic factors to facilitate pre-operative decision-making in patients considered candidates for pancreatic enucleation. Firstly, we utilized a multi-institution global cohort to capture and evaluate risk factors associated with post-operative complications after pancreatic enucleation; secondly, we conducted a single-institutional study to identify factors that increased the likelihood for developing a POPF from our experience that includes demographic, surgical and pathologic variables plus the impact of lesion distance from the main pancreatic duct (MPD).

## Methods

### Study design

**Fig. 1:**
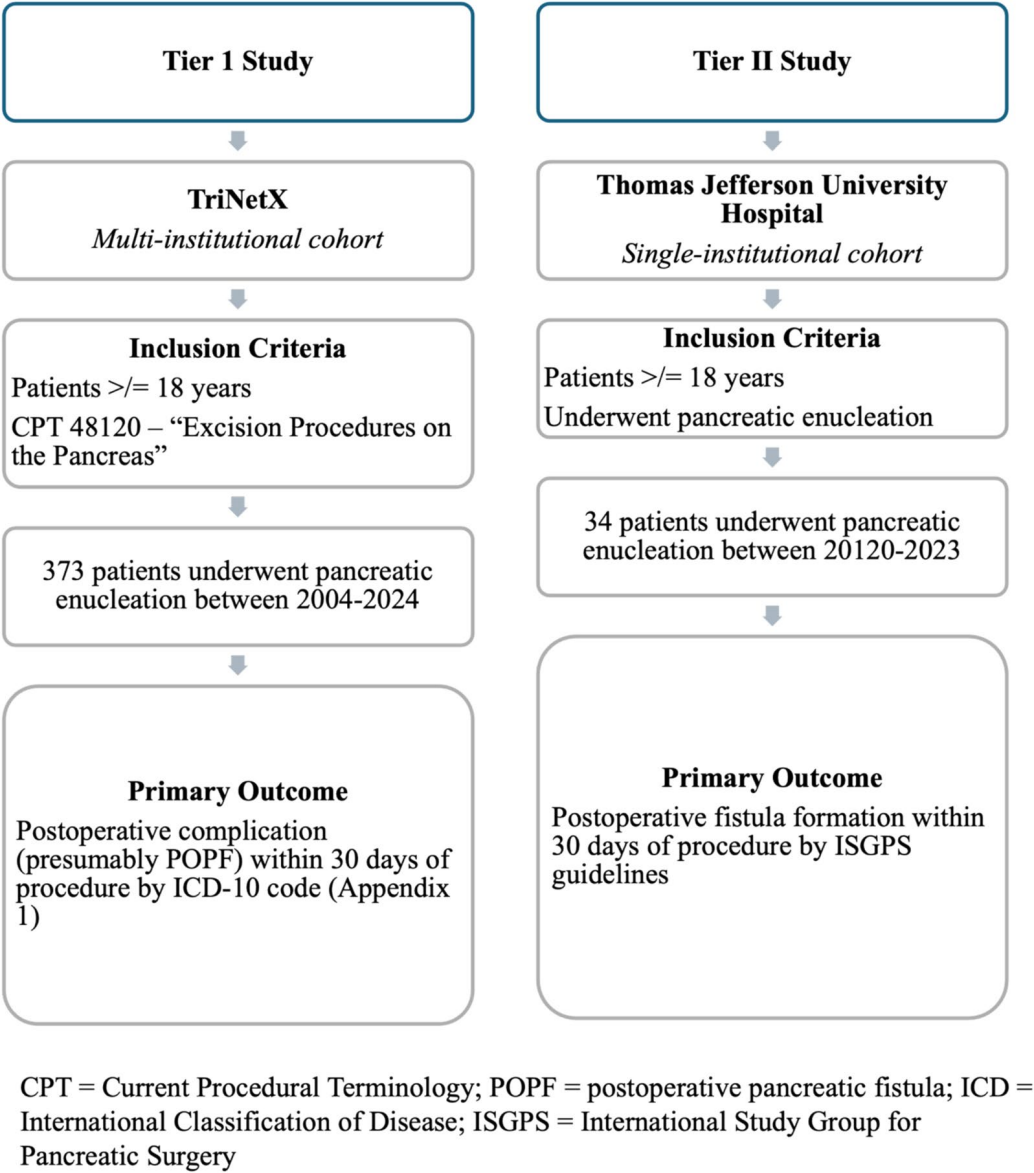
Tier I and Tier II study design

This was a two-tiered retrospective, exploratory study, which includes patients derived from two sources: (1) a multi-institutional research network, TriNetX and (2) a single-institutional cohort at the Thomas Jefferson University Hospital (TJUH) as shown in Fig. 1.

#### TriNetX database (Tier I study)

TriNetX is a multinational research network that aggregates de-identified patient data from multiple healthcare organizations. Our inclusion criteria required patients ≥ 18 years of age who underwent pancreatic enucleation based on CPT code 48,120 – “Excision Procedures on the Pancreas” between 2004–2025. Patient-level demographic and clinical factors examined included age, sex, race, body mass index (BMI), history of chronic kidney disease (CKD), coronary artery disease (CAD), congestive heart failure (CHF), chronic obstructive pulmonary disease (COPD), hypertension (HTN), hyperlipidemia (HLD), non-alcoholic fatty liver disease (NAFLD), acute and chronic pancreatitis, gastroesophageal reflux disease (GERD), nicotine dependence, type 2 diabetes mellitus (T2DM), deep vein thrombosis (DVT), pulmonary embolism (PE), thrombophlebitis, ascites and cirrhosis. The identified pre-operative comorbidities required documentation within 30 days of the procedure. The primary outcome was the occurrence of post-operative complications within 30 days of surgery as coded by specified ICD-10 codes. ICD-10 codes associated with post-operative complications related to this pancreatic procedure are shown in Appendix 1 and are as follows: K63.0—"Abscess of intestine"; K65.1—"Peritoneal abscess"; K68.1—"Retroperitoneal abscess"; K86.89—"Other specified diseases of the pancreas”; T81.4—"Infection following a procedure"; 0W9G—"Anatomical Regions, General / Drainage / Peritoneal Cavity"; 0D9W—"Gastrointestinal System / Drainage / Peritoneum"; 1,021,538—"Image-guided fluid collection drainage by catheter (e.g., abscess, hematoma, seroma, lymphocele, cyst)". POPF was not directly captured as there is not a specific ICD-10 code for pancreatic or pancreaticoduodenal fistulas.

#### Single institution database (Tier II study)

The single-institutional cohort was sourced from an institutional review board approved, prospectively maintained pancreatic surgery database at TJUH. Patients who underwent pancreatic enucleation from January 1, 2012, to August 14, 2023, were reviewed. Inclusion criteria required patients ≥ 18 years of age who underwent pancreatic enucleation. Patient demographic factors examined included age, sex, BMI, smoking status, history of diabetes and pancreatitis. Clinicopathologic characteristics studied incorporated tumor-specific characteristics such as tumor size and histopathology as well as radiologic imaging performed (multidetector computed tomography [CT/MDCT] and T1/T2 magnetic resonance cholangiopancreatography [MRI/MRCP]), type of surgery, length of stay (LOS), and estimated blood loss (EBL). Specifically, MDCT and MRCP imaging employed a dedicated pancreas protocol to assess and measure the shortest distance between the pancreatic neoplasm and the main pancreatic duct (MPD). The imaging modality performed was per provider preference. For MDCT, thin slice high-resolution (axial and coronal) contrast enhanced and reformatted imaging of the pancreas provided optimal spatial and detailed anatomic visualization to calibrate and measure the distance between the neoplasm and the MPD. MRCP provided enhanced visualization of the pancreatic duct by utilizing T2 weighted imaging sequences that are highly sensitive to fluid/pancreatic secretions to measure the distance of the MPD from the neoplasm. Of note, endoscopic ultrasound was not used to measure distance of the MPD from the neoplasm in this study. The primary outcome variable was development of POPF within 30 days of enucleation. We utilized the standard International Study Group of Pancreatic Fistula (ISGPF) classification to define the severity of the POPF [7].

### Statistical analysis

Descriptive and analytic statistics were performed using SPSS version 29. Mann–Whitney U test and odds ratios using 95% confidence interval were calculated to examine relationships between demographic and clinicopathologic variables and clinical outcomes as appropriate. Multivariate analysis was performed from a Cox proportional hazards regression model to identify independent predictors of clinical outcomes, with results reported as hazard ratios and corresponding 95% confidence intervals. Of note, TriNetX did not allow inclusion of BMI in the multivariable model. A two-sided *p*-value < 0.05 was used to determine significance in all analyses. Predictive accuracy for risk of a POPF occurring was predicated on measuring the neoplasm distance from the MPD assessed using receiver operator characteristics and calculating the area under the curve. In addition, likelihood ratios, sensitivity and specificity were calculated at predefined distance thresholds to evaluate discriminative ability of MPD-neoplasm distance in predicting POPF risk across multiple cutoff points.

## Results

### TriNetX database (Tier I study)

We identified 423 patients who underwent pancreatic enucleation between 2004 and 2025. There were 191 (45.2%) male patients. Mean patient age was 55.0 years (standard deviation [SD] = 18.2). Most patients were identified as White/Caucasian (*n* = 301, 71.2%). Of the cohort, 128 patients experienced post-operative complications after pancreatic enucleation (30.3%).

#### TriNetX database univariate analysis

Patients with history of HLD (OR = 2.37, CI: 1.29–4.35, *p* < 0.01), GERD (OR = 3.87, CI: 2.30–6.53, *p* < 0.001), nicotine dependence (OR = 2.36, CI: 1.11–5.01, *p* < 0.01), ascites (OR = 6.49, CI: 1.69–24.88, *p* < 0.01), DVT, PE, and thrombophlebitis (OR = 2.95, CI: 1.24–7.01, *p* < 0.05), within 30 days of the procedure were more likely to suffer from post-operative complications on univariate analysis. Additionally, patients with acute pancreatitis (OR = 8.28, CI: 3.61–19.01, *p* < 0.001), chronic pancreatitis (OR = 4.76, CI: 1.96–11.54, *p* < 0.001) within 30 days of the procedure were also more likely to undergo complications after pancreatic enucleation. Moreover, patients with BMI ≥ 25 (OR = 1.56, CI: 1.02–2.38, *p* < 0.05) were also higher risk for post-operative complications. Notably, risk factors such as race/ethnicity, and history of CKD, CAD, CHF, COPD, HTN, NAFLD, type 2, diabetes mellitus and cirrhosis did not reach statistical significance (Table 1).

**Table 1:** Preoperative clinicopathologic characteristics, demographic and clinical outcomes from Tier I- TriNetX cohort

Clinicopathologic Characteristics & Demographics	Overall (*N* = 423)	Patients with complications (*N* = 128)	Patients without complications (*N* = 295)	Odds Ratios (95% CI)	*P* Value	Hazard Ratio (95% CI)	*P* Value
Age at surgery: years (SD)	55.0 (18.2)	53.7 (16.0)	55.6 (18.2)		0.321	0.989 (0.981- 0.997)	< 0.01
Male (%) Female (%) Unknown (%)	191 (45.2%) 225 (53.2%) 7 (1.7%)	57 (44.5%) 67 (52.3%) 4 (3.2%)	134 (45.4%) 158 (53.6%) 3 (1.0%)		0.296	1.32 (1.04- 1.89)	0.052
Race / Ethnicity (%) *White/Caucasian	301 (71.2%)	94 (73.4%)	207 (70.2%)	1.18 (0.74—1.87)	0.496		
CKD (%)	14 (3.3%)	3 (2.3%)	11 (3.7%)	0.62 (0.17—2.26)	0.468		
CAD (%)	32 (7.6%)	13 (10.2%)	19 (6.4%)	1.64 (0.78—3.44)	0.188		
CHF (%)	9 (2.1%)	2 (1.6%)	7 (2.4%)	0.65 (0.13—3.19)	0.598		
COPD (%)	7 (1.7%)	3 (2.3%)	4 (1.4%)	1.75 (0.39—7.92)	0.470		
HTN (%)	31 (7.3%)	8 (6.3%)	23 (7.8%)	0.59 (0.21—1.63)	0.309		
HLD (%)	48 (11.3%)	23 (18.0%)	25 (8.5%)	2.37 (1.29—4.35)	< 0.01	0.62 (0.32–1.21)	0.985
NAFLD (%)	2 (0.5%)	1 (0.9%)	1 (0.4%)	2.31 (0.14—37.30)	0.554		
**BMI at surgery (SD) BMI ≥ 25 (%) BMI < 25(%)	28.3 (6.9) 236 (55.7%) 187 (44.3%)	28.1 (7.0) 81 (63.3%) 47 (37.7%)	28.4 (6.9) 155 (52.5%) 140 (47.5%)	1.56 (1.02 – 2.38)	0.325 < 0.05		
Chronic pancreatitis (%)	23 (5.4%)	15 (11.7%)	8 (2.7%)	4.76 (1.96—11.54)	< 0.001	1.78 (1.21–2.55)	< 0.005
Type 2 diabetes mellitus (%)	47 (11.1%)	20 (15.6%)	27 (9.2%)	1.84 (0.99—3.42)	0.054		
GERD (%)	73 (17.3%)	41 (32.0%)	32 (10.8%)	3.87 (2.30—6.53)	< 0.001	1.07 (0.75–1.52)	0.726
Nicotine Dependence (%)	26 (6.1%)	15 (11.7%)	11 (3.7%)	2.36 (1.11—5.01)	< 0.01	1.06 (0.66- 1.72)	0.803
DVT, PE and thrombophlebitis (%)	22 (5.2%)	12 (9.4%)	10 (3.4%)	2.95 (1.24—7.01)	< 0.05	1.74 (1.02–2.99)	< 0.05
Acute Pancreatitis (%)	32 (7.6%)	24 (18.8%)	8 (2.7%)	8.28 (3.61—19.01)	< 0.001	1.64 (1.07–2.51)	< 0.05
Ascites (%)	11 (2.6%)	8 (6.3%)	3 (1.0%)	6.49 (1.69—24.88)	< 0.01	2.96 (1.70–5.15)	< 0.001
Cirrhosis (%)	18 (4.3%)	6 (4.7%)	12 (4.1%)	1.16 (0.43—3.16)	0.772		

^*^Due to limitation of the TriNetX database, additional race/ethnicity data was unavailable if there were less than 10 patients in the cohort

^**^Multivariate analysis function was unavailable for BMI from the TriNetX database

*BMI* body mass index, *CKD* chronic kidney disease, *CAD* coronary artery disease, *CHF* congestive heart failure, *COPD* chronic obstructive pulmonary disease, *DVT* deep vein thrombosis, *GERD* gastroesophageal reflux disease, *HTN* hypertension, *HLD* hyperlipidemia, *NAFLD* non-alcoholic fatty liver disease, *OR* odds ratio, *PE* pulmonary embolism, *POPF* post-operative pancreatic fistula, *SD* standard deviation

#### TriNetX database multivariate analysis

Demographic characteristics and risk factors identified on univariate analysis as significant for post-operative complications after pancreatic enucleation were included in the multivariable model. Of note, TriNetX did not allow for BMI to be included in the multivariate analysis; therefore, BMI was excluded from the multivariate analysis although it was a significant risk factor for post-operative complications on univariate analysis. Multivariate analysis showed patients with acute pancreatitis (HR = 1.64, CI: 1.07–2.51, *p* < 0.05), chronic pancreatitis (HR = 1.78, CI: 1.21–2.55, *p* < 0.005), ascites (HR = 2.96, CI: 1.70–5.15, *p* < 0.001), and DVT, PE and thrombophlebitis (HR = 1.74, CI: 1.02–2.99, *p* < 0.05) within 30 days of enucleation were more likely to undergo post-operative complications. Furthermore, advanced age was associated with a slightly reduced risk of post-operative complications (HR = 0.99, CI: 0.981–0.997, *p* < 0.01). In contrast, HLD, GERD, and nicotine dependence was no longer significantly associated with increased risk for post-operative complications (Table 1).

### Single institution database (Tier II study)

We identified 34 patients who underwent pancreatic enucleation between 2012 and 2023. There were 12 male patients (35.2%). Mean patient age was 57.3 years (SD = 13.5). Most patients were identified as White/Caucasian (*n* = 30, 88.2%). Mean tumor size was 2.5 cm (SD = 1.43). Neoplasms that underwent pancreatic enucleation were classified on final histopathology as follows: neuroendocrine tumor (NET) (*n* = 18, 53%), among these, sixteen (88.9%) were well-differentiated NET; Other benign’ lesions (*n* = 8, 23.5%), intraductal papillary mucinous neoplasms (IPMN) (*n* = 4, 11.8%), insulinoma (*n* = 2, 5.9%), and malignancy (*n* = 2, 5.9%). Of the two patients with malignancy, one patient had metastatic NET, and one patient had a solid pseudopapillary neoplasm. Mean body mass index was 27.9 (SD = 4.5). Most patients underwent open pancreatic enucleation (*n* = 24, 70.6%), followed by robotic (*n* = 5, 14.7%) and laparoscopic pancreatic enucleations (*n* = 5, 14.7%). The mean anteroposterior (AP) diameter of the pancreatic body was 23.2 mm (mm) (SD = 4.4). Mean distance from lesion to MPD was 5.4 mm (SD = 4.5) (Table 2). EBL was 25 mL (IQR: 100 days) and median LOS was 3 days (interquartile range [IQR]: 5 days).

**Table 2:** Preoperative clinicopathologic characteristics, demographic and clinical outcomes from Tier II -Single-Institutional cohort

Clinicopathologic Characteristics & Demographics	Overall	POPF (*N* = 8)	Non POPF (*N* = 26)	Odds Ratio (95% CI)	Mann–Whitney U Statistic	*P* Value
Age at surgery: years (SD)	57.3 (13.5)	58.7 (11.2)	57.0 (14.1)			0.759
Male (%)	12 (35.3%)	4 (50%)	8 (30.9%)	0.456 (0.066–3.111)		0.410
BMI (SD)	27.8 (4.5)	29.5 (6.2)	27.4 (3.9)			0.257
Race/Ethnicity (%)						0.098
White/Caucasian	30 (88.2%)	6 (75%)	24 (92.3%)			
Black	2 (5.9%)	2 (25%)	0 (0%)			
Hispanic	0 (0%)	0 (0%)	0 (0%)			
API	2 (5.9%)	0 (0%)	2 (7.7%)			
Other	0 (0%)	0 (0%)	0 (0%)			
Current/Former Smoker	7 (20.6%)	3 (37.5%)	4 (15.4%)	0.316 (0.038- 2.842)		0.315
Grade of POPF						
Grade A (biochemical leak)		4 (50%)				
Grade B		3 (37.5%)				
Grade C		1 (12.5%)				
Tumor Size (cm)	2.5 (1.4)	2.4 (1.1)	2.6 (2.1)			0.778
Tumor Histopathology (%)						0.684
Benign NET	18 (53%)	6 (75%)	12 (46.2%)			
Well-differentiated	16 (88.9%)	6 (100%)	10 (83.3%)			
Other benign (cyst, etc.)	8 (23.5%)	0 (0%)	8 (30.8%)			
IPMN	4 (11.8%)	2 (33%)	2 (7.7%)			
Insulinoma	2 (5.9%)	0 (0%)	2 (7.7%)			
Malignancy	2 (5.9%)	0 (0%)	2 (7.7%)			
Metastatic NET	1 (50%)		1 (50%)			
SPN	1 (50%)		1 (50%)			
Tumor location (%)						0.1863
Head	12 (35.3%)	2 (25%)	8 (30.7%)			
Body	10 (29.4%)	2 (25%)	8 (30.7%)			
Tail	10 (29.4%)	2 (25%)	10 (38.4%)			
Head/body	1 (2.9%)	1 (12.5%)	0 (0%)			
Body/tail	1 (2.9%)	1 (12.5%)	0 (0%)			
Distance from lesion to MPD (mm) (SD)	4.9 (4.3)	2.8 (2.6)	6.5 (4.5)			0.05
AP diameter of pancreatic body (mm) (SD)	23.2 (4.4)	25.1 (4.7)	22.4 (4.1)			0.982
Length of stay (days) (IQR)	3 (5)	5.5 (3.5)	3 (1.3)		12.66	0.01
Estimated blood loss (mL) (IQR)	25 (100)	175 (225)	43.5 (82.5)		4.94	0.05
History of chronic pancreatitis	3 (8.8%)	2 (25%)	1 (3.8%)	0.120 (0.009–1.553)		0.105
History of Type 2 diabetes mellitus	3 (8.8%)	1 (12.5%)	2 (9%)	0.594 (0.027- 39.279)		1.00
Surgical approach						0.326
Laparoscopic	3 (8.8%)	1 (12.5%)	2 (7.7%)			
Robotic	7 (20.6%)	3 (37.5%)	4 (15.4%)			
Open	24 (70.6%)	4 (50%)	20 (77%)			

*SD* standard deviation, *BMI* body mass index, *IQR* interquartile range, *NET* neuroendocrine tumor, *SPN* solid pseudopapillary neoplasm, *MPD* main pancreatic duct, *OR* odds ratio, *POPF* post-operative pancreatic fistula

**Fig. 2:**
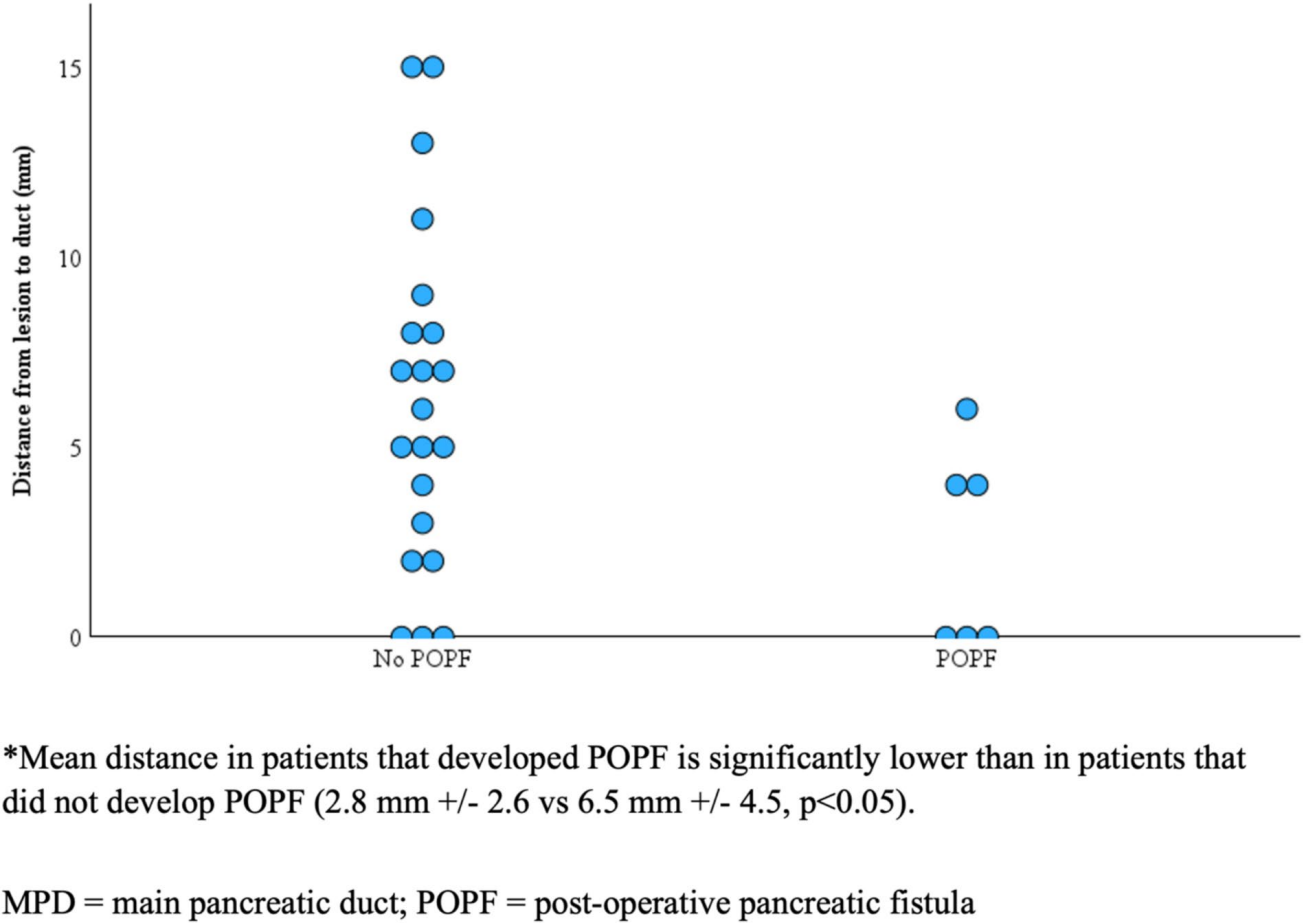
Distance from MPD to lesion in POPF and non-POPF Patients*

POPF was noted in 8 patients (23.5%). The mean age was 58.7 years (SD = 11.2). There were 4 male patients (50%). The mean BMI was 29.5 (SD = 6.2) and mean tumor size was 2.4 cm (SD = 1.1) with the majority being well-differentiated neuroendocrine tumors on final pathology (*n* = 6, 75%). Of the 8 patients who developed POPF, half were defined as ISGPS grade A (biochemical leak) (*n* = 4, 50%); the remaining were ISGPS grade B (*n* = 3, 37.5%) and grade C (*n* = 1, 12.5%). Among patients who developed POPF, the mean distance from the lesion to MPD was 2.8 mm (SD = 2.6) compared to 6.5 mm (SD = 4.5) in patients who did not develop a POPF (*P* < 0.05). The Receiver Operating Characteristic-Area Under Curve (ROC-AUC) for this metric demonstrated an area of 0.795 (*p* < 0.005) (Fig. 2, Supplementary Fig. 1). Patients who developed POPF had a significantly higher EBL (175 vs 43.5 mL; *p* < 0.05) and LOS (5.5 vs 3 days; *p* < 0.01). There was no significant difference in age, sex, race/ethnicity, BMI, smoking status, tumor size or location, tumor histopathology, surgical approach, diameter of MPD, history of pancreatitis, or history of type 2 diabetes mellitus between patients who had occurrence of POPF versus patients who did not (Table 2).

## Discussion

Although pancreatic enucleation for benign and low-risk tumors is parenchymal sparing and preserves exocrine and endocrine pancreatic functions, it is associated with high rates of post-operative complications, and specifically post-operative fistula formation as shown herein. Identifying risk factors attributable to frequently occurring complications, including POPFs, after pancreatic enucleation is warranted. The purpose of this study is three-fold. Firstly, to examine the risk factors associated with post-operative complications after pancreatic enucleation derived from a large multi-institutional cohort of patients. Secondly, to identify clinicopathologic, histologic and radiologic factors implicated with POPF from a well-characterized, prospectively maintained, single-institution cohort of patients post enucleation. Lastly, to provide surgeons with a summative strategy based upon this data and our experience to aid in the selection of patients most suitable for pancreatic enucleation.

### Tier I study: TriNetX database

The TriNetX database is a large global platform and provides a cross-sectional assessment of risk factors associated with post-operative complications after pancreatic enucleation. TriNetX does not specifically report on the occurrence of POPFs, but summative coded associated complications related to this procedure have been included (Appendix I). Within this large multi-institutional cohort of patients, we identified 30.3% of patients who developed post-operative complications after pancreatic enucleation. Although we cannot comment on the specific occurrence of POPF in the TriNetX cohort, POPF is the most commonly occurring complication among patients who undergo pancreatic enucleation as documented in the literature. Briefly, Zhang et al. studied 119 patients who underwent pancreatic enucleation between 2005 and 2011 with an overall morbidity rate of 67.2%. Clinically significant POPF (grades B and C) was the most common complication in 28% of patients [8]. Likewise, Strobel et al. reported that POPF was the ‘main determinant of outcome’ in 41% of patients [9]. Crippa et al. also observed a 43% POPF rate among 61 patients, along with Brient et al. reporting POPF occurring in 27% of their patients [6, 10]. Xu et al. similarly reported upon clinically relevant POPFs occurring in 43% of 81 patients who underwent laparoscopic pancreatic enucleation [11]. These formidable rates of morbidity (majority from POPF) are well within the range of overall complication rates reported in the TriNetX cohort.

Using the TriNetX platform, we subsequently identified risk factors associated with complications after enucleation. Among these, patients with a BMI ≥ 25 were one and a half times more likely to develop post-operative complications as compared to patients with BMI ≤ 25. In a study by Jilesen et al., BMI ≥ 25 was reported to be a risk factor associated with post-operative complications after pancreatic tumor enucleation [12]. In a recent study by Vlad et al., incidence of POPF was notably doubled in patients with obesity who underwent pancreatic enucleation [13]. This finding supports previous observations with higher occurrences of complications after pancreatic surgery in the overweight or morbidly obese patient [14]. Interestingly, we also found that patients with HLD were more than 2 times more likely to experience post-operative complications, which is likely given HLD’s association with elevated BMI. Studies have shown 60–70% patients with obesity have dyslipidemia [15]. Although BMI was excluded in the multivariable model due to limitations of the TriNetX platform, HLD was not identified as a significant risk factor on multivariate analysis.

In this study, we also found patients with preoperative diagnosis of acute and chronic pancreatitis that had a significantly higher risk of developing post-operative complications on univariate and multivariate analysis. As supported in the literature, Strobel et al. and Wang et al. identified acute pancreatitis as an independent risk factor for POPF after pancreatic enucleation [9, 16]. Likewise, Callery et al. described the soft pancreatic parenchyma, which is found in acute pancreatitis, also a risk factor for development of POPF. There are many explanations for this association. For example, soft pancreatic parenchyma may be more susceptible to ischemia and injury in part attributable to the preserved exocrine function, increased secretion of caustic pancreatic enzymes with inherent local and systemic effects [17]. Patients with chronic pancreatitis were also found to be more susceptible to suffer from post-operative complications after enucleation. In a study by Distler et al., chronic pancreatitis of the pancreatic remnant was identified as an independent risk factor for development of POPF in patients who underwent distal pancreatectomy. This may be due to chronic inflammation and ductal stenosis [18]. Although much of the current literature studies the association between pancreatitis and POPF in patients who undergo traditional pancreatic resection, underlying mechanisms likely apply to pancreatic enucleation. Our study shows significant associations between acute and chronic pancreatitis and post-operative complications in pancreatic enucleation.

Our study also found associations between GERD, DVT, PE and thrombophlebitis, ascites, and nicotine dependence with post-operative complications or POPF after pancreatic enucleation on univariate analysis. However, multivariable analysis identified only DVT, PE, and thrombophlebitis and ascites as independent risk factors. Currently, limited literature exists studying the association between GERD, DVT, PE and thrombophlebitis, ascites, and nicotine dependence with post-operative complications or POPF after pancreatic enucleation. To our knowledge, our study is the first to highlight such associations. These findings may in part be attributed to these individual comorbidities. However, it is more likely the cumulative result of suboptimal physiological and performance parameters as defined by the Eastern Cooperative Oncology Group (ECOG) ≥ 3; in these patients what we strive to optimize prior to recommending pancreatic resection [19]. Specifically, with regards to GERD, this may contribute to poor oral intake and thereby suboptimal pre-operative nutrition. Poor nutritional status is a well-studied risk factor for post-operative complications in the gastrointestinal cancer literature [20–22]. In a study of 1,420 general surgery patients, 30-day readmission was highest in patients who underwent pancreatic surgery; compromised nutritional status was a frequent reason [23]. In another study evaluating patients who underwent surgery for pancreatic ductal adenocarcinoma (PDAC), a low prognostic nutrition index was associated with increased post-operative complications, specifically POPF [24]. Moreover, Enhanced Recovery After Surgery (ERAS) Society guidelines include perioperative oral immune-nutrition to decrease risk of infectious complications in pancreaticoduodenectomy patients [25]. Notably, we found that patients with history of DVT, PE and thrombophlebitis were significantly more likely to develop post-operative complications in unadjusted and adjusted models. Indeed, it is plausible as these patients are at a higher risk for post pancreatectomy hemorrhage which is a complication that can also exacerbate pancreatic fistula formation [26]. This association has yet to be elucidated in the current literature after pancreatic enucleation. Further investigation appears warranted to explore this relationship. In addition, patients with a preoperative presence of ascites had a higher likelihood of having postoperative complications after enucleation in univariate and multivariate models. While there has not been direct evidence linking preoperative ascites to increased risk of POPF, ascites is often associated with liver cirrhosis, portal hypertension and suboptimal ECOG performance status: each attributable to a higher risk for morbidity after surgery, in this case, pancreatic enucleation. Due to limitations of the TriNetX platform, we were unable to determine how many patients with preoperative ascites had preoperative cirrhosis; however, patients with preoperative cirrhosis were not significantly more likely to develop post-operative complications. We also found nicotine dependence increased the likelihood of postoperative complications in patients who underwent enucleation; however, this was not an independent risk factor on multivariate analysis. Studies have shown smoking status as an independent predictor of POPF following pancreatic surgery [27, 28]. Further research is necessary to better elucidate this association in patients who underwent pancreatic enucleation.

### Tier II study: single institution database

Within the tier II single-institutional cohort, we identified 23.5% of patients who experienced POPF after pancreatic enucleation. This finding is consistent with the incidence of POPFs reported in the literature[7]. Likewise, as reported by other investigators, we did not observe an association between tumor size, tumor histopathology, pancreatic texture, or parenchymal anteroposterior (AP) diameter with fistula formation after pancreatic enucleation in our study group [8, 29]. Notably, our patients with POPFs had a significantly higher intraoperative blood loss.

Importantly, we identified distance from the MPD to the pancreatic lesion as a significant determinant for the development of a POPF. Specifically, we found the distance from the neoplasm to the main pancreatic duct (MPD) was significantly less in patients who developed POPF (2.8 mm ± 2.6) compared to those who did not (6.5 mm ± 4.5) and was highly predictive for POPF. This finding supports previous studies that propose a distance of at least 2 to 3 mm from the MPD as safe to proceed with pancreatic enucleation [30, 31]. Notably, few studies specifically explored the actual ‘measured proximity’ of lesions to the MPD as a risk factor for POPF utilizing preoperative imaging studies [5, 6]. From these reports, the data remains discordant. Zhang et al. identified risk factors associated with POPF, however, the main limitation of their study was that the distance between the lesion and MPD was unable to be analyzed and therefore not assessed routinely [8]. Jin et al. retrospectively studied 56 patients who underwent open and robotic pancreatic enucleation in China identifying a distance between the MPD and lesion as ≤ 4.5 mm in patients who developed POPF versus ≥ 5 mm in patients who did not (*p* < 0.05) based on preoperative radiologic imaging [32]. Brien et al. later studied 52 patients who underwent pancreatic enucleation in a French hospital multicenter study and noted that a ≤ 2 mm distance between the tumor and MPD promotes occurrence of POPF. These authors also tested a 3- and 4 mm cutoffs that was not found to be statistically significant [6]. Heeger and colleagues divided patients into two study groups: either deep enucleation (distance from the lesion to the MPD ≤ 3 mm) or standard enucleation (distance from lesion to the MPD ≥ 3 mm) determined by intraoperative ultrasound. They found POPF rates significantly higher after pancreatic enucleation for tumors located within 3 mm of the MPD [5]. Interestingly, both study designs proposed binary cut-offs for distance from MPD to assess POPF occurrence; however, they did not identify other concordant cutoffs with associated POPF risk and therefore could not assess the risk of POPFs above or below their suggested distances. Lastly, Strobel et al. suggested no association between distance between the lesion and the MPD and the occurrence of a POPF. However, the authors acknowledged that this may have been due to 98.2% of patients undergoing open pancreatic enucleation. This finding is dissimilar to our tier II study cohort where the majority of patients (70.6%) underwent open pancreatic enucleation with no apparent impact on fistula formation [9].

Interestingly, our analysis further suggests a potential continuous relationship between the radiologic distance from the MPD and formation of POPF, rather than a binary cut-off. Additional analysis including optimal positive likelihood ratios (LR +) and Chi-square analysis at specific distances from the MPD has been provided as Supplementary Table 1 and 2. The optimal LR + occurs at 1.0 mm (LR + = 3.50) and 4.5 mm (LR + = 2.50) while balancing both sensitivity and specificity. Moreover, 4 mm and 6 mm were identified as statistically significant associations between distance from MPD and POPF formation based on Chi-square analysis and may represent clinically relevant thresholds for risk stratification. However, given the small sample (*n* = 8) for this analysis, we recommend further studies with larger sample sizes to allow for robust clinical applicability of this data. Recognizing this continuum as a risk-based model predicated on the neoplasm’s proximity to the MPD allows for individualized risk assessment(s) in the preoperative planning period and provides an important platform for continued study. Furthermore, the increase in LOS in the POPF cohort is expected, and likely due to the downstream outcome reflecting the morbidity related with POPF. This study contains several clear limitations, which may limit the external validity of our findings. The TriNetX database is a retrospective database and may introduce bias, specifically selection bias. The presumption that most post enucleation complications are attributable to POPF is greatly supported in the literature; however, this variable was not specifically categorized as such in TriNetX reporting. Therefore, we were limited by the variables captured by TriNetX. We do acknowledge that any disparities may be attributable to the inherent differences in sample sizes, as the current published experience is limited to smaller, single-center studies compared to data captured in a multinational research consortium network. Another significant limitation of TriNetX was the aggregation of postoperative complications into a single category, which restricted the granularity of our analysis. Moreover, TriNetX does not specify patient and operative descriptors; thus, our analyses relied on generic ICD and CPT codes. We also were unable to obtain Clavien Dindo classifications from the TriNetX database to determine the severity of complications. Additionally, tumor-specific characteristics were not available. Moreover, our single-institution cohort was prospectively maintained but with a small sample size, which is another limitation. For example, our limited study cohort size precluded a targeted subgroup analyses exploring impact of technical considerations with enucleation (e.g. energy device versus dissection with ties and consequences of direct ligation of identified feeding branch pancreatic ducts versus not) on outcomes that represents an important platform for further study. Notably, CT and MRI do provide a reliable assessment evaluating lesion location and involvement with adjacent structures. However, we acknowledge that absence of endoscopic ultrasound to more accurately measure the distance between the tumor and the MPD is a methodological limitation as CT and MRI may have limited spatial resolution. Indeed, subsequent investigations with larger sample size and a prospective study design are necessary to determine if radiologic distance between the MPD and the lesion can be used as a validated tool to decrease POPF formation in patients who undergo pancreatic enucleation.

**Fig. 3:**
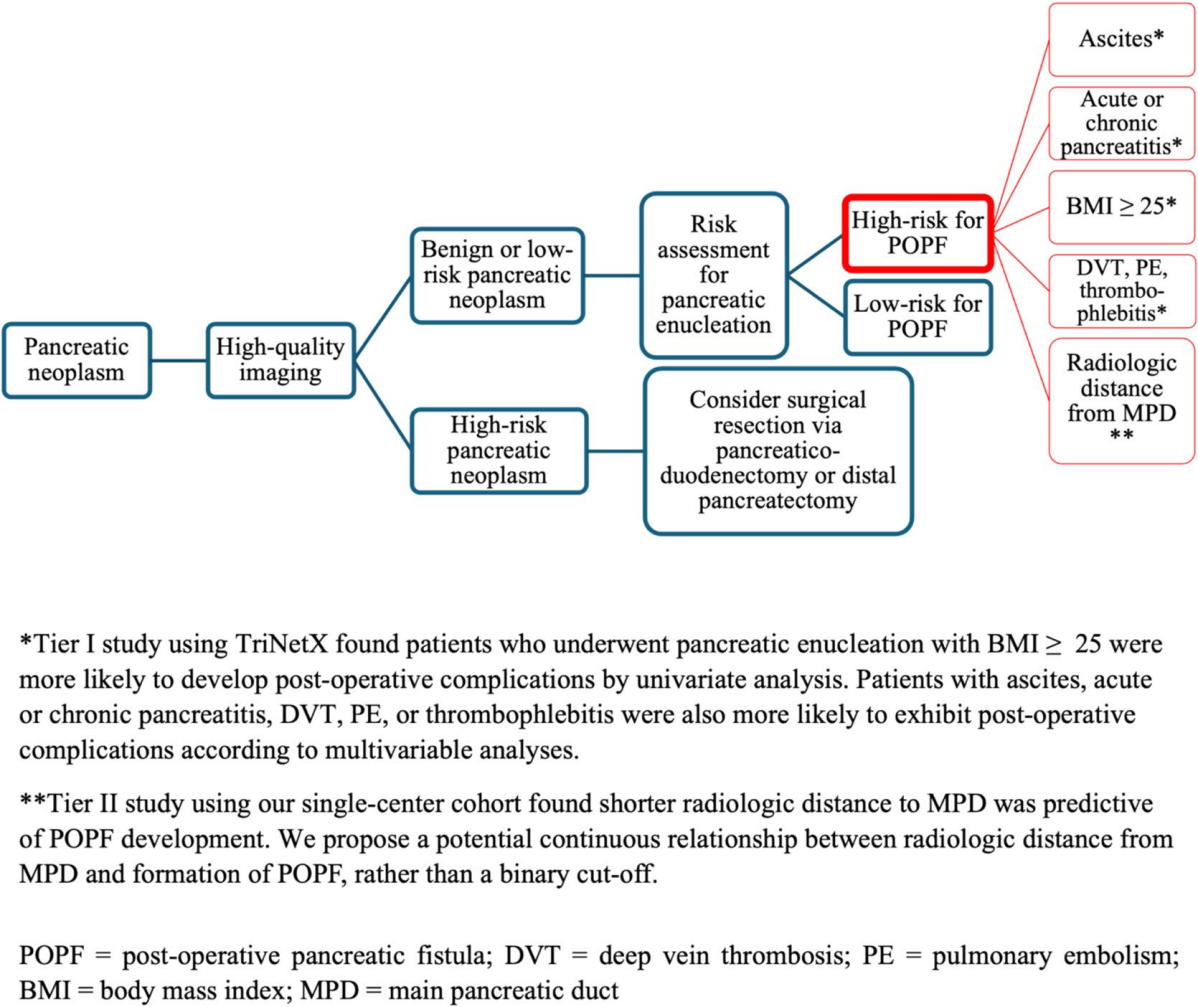
Algorithm for classification of patients with pancreatic tumors to identify high-risk features of post-operative fisutla formation

Given our study findings and experience at a high-volume pancreatic surgery center, we offer a summative pre-operative algorithm for surgeons and/or providers to help identify optimal candidates for pancreatic enucleation and minimize occurrence of POPFs (Fig. 3). Importantly, this strategy highlights the need for high-quality imaging via CT/MDCT or MRI/MRCP as part of the preoperative work-up and recommends a risk assessment for patients who may be candidates for pancreatic enucleation. We further propose stratifying patients as high-risk or low-risk for POPFs based on suggested demographic, clinical factors and measured radiologic distance from the MPD as identified in our two-tiered study. We believe this approach will allow for a more individualized, streamlined decision-making process between patient and surgeon when offering pancreatic enucleation to patients with benign or low-risk pancreatic tumors.

## Conclusions

This two-tiered study contributes to the current literature regarding commonly occurring POPFs after pancreatic enucleation. Our study design allowed us to evaluate risk factors associated with post-operative complications from two distinct sources: a multi-institutional health research network in combination with a more granular review from a single institution experience for risk factors associated with POPF. We identified acute and chronic pancreatitis, ascites, DVT, PE and thrombophlebitis on multivariate analysis, and BMI ≥ 25 on univariate analysis as risk factors associated with post-operative complications after pancreatic enucleation. More specifically, we identified radiologic distance between the MPD and the lesion as an important predictive factor for POPF formation. Looking ahead, this data will aid in identifying patients who are optimal candidates for pancreatic enucleation and allow for a more individualized approach in the preoperative decision-making for patients undergoing pancreatic enucleation.

## Supplementary Information

Supplementary Material 1.

## Data Availability

The datasets used and/or analyzed during the current study are available from the corresponding author on reasonable request.

## Abbreviations

NF-pNETS: Nonfunctioning pancreatic neuroendocrine tumors
SCN: Serous cystic neoplasms
MCN: Mucinous cystic neoplasms
SPCN: Solid papillary cystic neoplasms
BD-IPMN: Branch duct intraductal papillary mucinous neoplasms
MPD: Main pancreatic duct
POPF: Postoperative pancreatic fistula
TJUH: Thomas Jefferson University Hospital
BMI: Body mass index
CKD: Chronic kidney disease
CAD: Coronary artery disease
CHF: Chronic heart failure
COPD: Chronic obstructive pulmonary disease
HTN: Hypertension
HLD: Hyperlipidemia
NAFLD: Non-alcoholic fatty liver disease
GERD: Gastroesophageal reflux disease
DVT: Deep vein thrombosis
PE: Pulmonary embolism
EGD: Esophagogastroduodenoscopy
CT: Computed tomography
MRI: Magnetic resonance imaging
EBL: Estimated blood loss
SD: Standard deviation
CI: Confidence interval
ROC-AUC: Receiver operating characteristic-area under curve
