# Supplementary material for: Clinicopathologic risk factors for post-operative complications after enucleation of pancreatic neoplasms
Source: World J Surg Oncol. 2025 Jul 22;23:293. doi: 10.1186/s12957-025-03920-0 (PMC12281995; doi:10.1186/s12957-025-03920-0)
Supplement: Supplementary file 1 — Supplementary Material 1. [file 12957_2025_3920_MOESM1_ESM.docx]

**Supplementary Files (Supplementary Figure 1, Supplementary Table 1, Supplementary Table 2, and Appendix 1)**

Supplementary Figure 1. ROC Curve Assessing Predictive Ability of Distance from MPD for POPF Formation


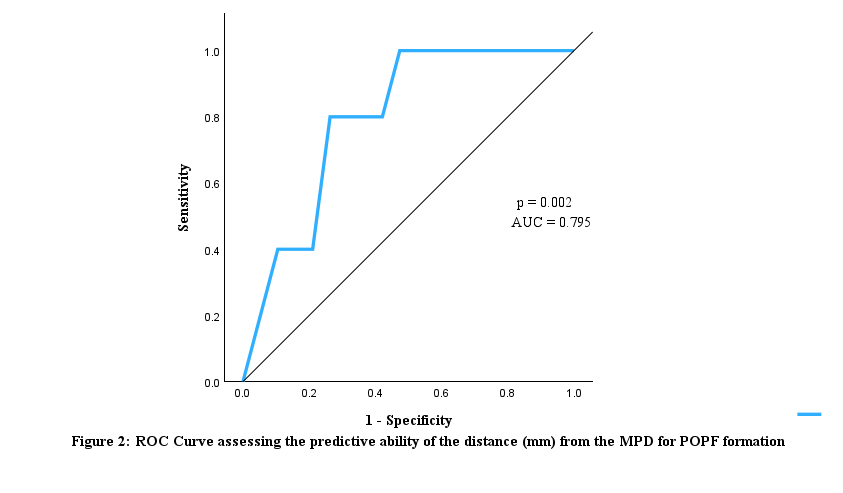


Supplementary Table 1. Sensitivity, Specificity, and Likelihood Ratios by Cutoff Distance from Main Pancreatic Duct

| Proposed Cutoff Distance from Main Pancreatic Duct (mm) | Sensitivity | 1 - Specificity | Positive Likelihood Ratio |
| --- | --- | --- | --- |
| 1.00 | 0.500 | 0.143 | 3.50 |
| 2.50 | 0.500 | 0.238 | 2.10 |
| 3.50 | 0.500 | 0.286 | 1.75 |
| 4.50 | 0.833 | 0.333 | 2.50 |
| 5.50 | 0.833 | 0.476 | 1.75 |
| 6.50 | 1.000 | 0.524 | 1.91 |
| 7.50 | 1.000 | 0.667 | 1.50 |
| 8.50 | 1.000 | 0.762 | 1.31 |
| 10.00 | 1.000 | 0.810 | 1.24 |

Supplementary Table 2. Association Between Cutoff Distance from Main Pancreatic Duct and Rate of Post-Operative Pancreatic Fistula Formation

| Threshold Distance from Main Pancreatic Duct (mm) | Chi-squared | P Value |
| --- | --- | --- |
| 1 | 2.608 | 0.106 |
| 2 | 1.434 | 0.231 |
| 3 | 1.001 | 0.317 |
| 4 | 4.242 | 0.039 |
| 5 | 2.380 | 0.123 |
| 6 | 4.359 | 0.037 |
| 7 | 2.712 | 0.100 |
| 8 | 1.804 | 0.179 |
| 9 | 1.395 | 0.238 |
| 10 | 1.395 | 0.238 |

**Appendix**

Appendix 1. ICD-10 Codes Associated with Post-Operative Complications Related to Pancreatic Procedures

1. ICD-10 K63.0 - "Abscess of intestine"
2. ICD-10 K65.1 - "Peritoneal abscess"
3. ICD-10 K68.1 - "Retroperitoneal abscess"
4. ICD-10 K86.89 - "Other specified diseases of the pancreas"
5. ICD-10 T81.4 - "Infection following a procedure"
6. ICD-10 0W9G - "Anatomical Regions, General / Drainage / Peritoneal Cavity"
7. ICD-10 0D9W - "Gastrointestinal System / Drainage / Peritoneum"
8. ICD-10 1021538 - "Image-guided fluid collection drainage by catheter (e.g., abscess, hematoma, seroma, lymphocele, cyst)"
